# Developing Transferable Fourier Transform Mid-Infrared Spectroscopy Predictive Models for Buffalo Milk: A Spatio-Temporal Application Strategy Analysis Across Dairy Farms

**DOI:** 10.3390/foods14060969

**Published:** 2025-03-12

**Authors:** Han Jiang, Peipei Wen, Yikai Fan, Yi Zhang, Chunfang Li, Chu Chu, Haitong Wang, Yue Zheng, Chendong Yang, Guie Jiang, Jianming Li, Junqing Ni, Shujun Zhang

**Affiliations:** 1 Key Lab of Agricultural Animal Genetics, Breeding and Reproduction of Ministry of Education, Huazhong Agricultural University, Wuhan 430070, China; whjianghan@wh.cebbank.com (H.J.); wenpeipei@webmail.hzau.edu.cn (P.W.); fanyikai123@webmail.hzau.edu.cn (Y.F.); zhangyi971115@163.com (Y.Z.); chunfangli0521@126.com (C.L.); chu1999@webmail.hzau.edu.cn (C.C.); htw0411@webmail.hazu.edu.cn (H.W.); zhengy@webmail.hzau.edu.cn (Y.Z.); 2 Frontiers Science Center for Animal Breeding and Sustainable Production, Huazhong Agricultural University, Ministry of Education, Wuhan 430070, China; 3 The Hebei Provincial Station for Livestock Varieties Producing and Spreading, Shijiazhuang 050061, China; dhihebei@163.com (C.Y.); 13833127625@163.com (G.J.); li13785153452@163.com (J.L.)

**Keywords:** buffalo milk, Fourier transform mid-infrared spectroscopy, modeling applications, spatio-temporal effects, nutritional quality parameters

## Abstract

A robust model of buffalo milk based on Fourier Transform Mid-Infrared Spectroscopy (FT-MIRS) is lacking and is difficult to complete quickly. Therefore, this study used 614 milk samples from two buffalo farms from south and central China for FT-MIRS to explore the potential of predicting buffalo milk fat, milk protein, and total solids (TS), providing a rapid detection technology for the determination of buffalo milk composition content. It also explored the rapid transformation and application of the model in spatio-temporal dimensions, providing reference strategies for the rapid application of new models and for the establishment of robust models. Thus, a large number of phenotype data can be provided for buffalo production management and genetic breeding. In this study, models were established by using 12 pre-processing methods, artificial feature selection methods, and partial least squares regression. Among them, a fat model with PLSR + SG (w = 15, *p* = 4) + 302 wave points, a protein model with PLSR + SG (w = 7, *p* = 4) + 333 wave points, and a TS model with PLSR + None + 522 wave points had the optimal prediction performance. Then, the TS model was used to explore the application strategies. In temporal dimensions, the TS model effectively predicted the samples collected in a contemporaneous period (RPD_V_ (Relative Analytical Error of Validation Set) = 3.45). In the spatial dimension, at first, the modeling was conducted using the samples from one farm, and afterward, 30–70% of a sample from another farm was added to the debugging model. Then, we found that the predictive ability of the samples from the other farm gradually increased. Therefore, it is possible to predict the composition of buffalo milk based on FT-MIRS. Moreover, when using the two application strategies that predicted contemporaneous samples as the model, and adding 30–70% of the samples from the predicted farm, the model application effect can be improved before the robust model has been fully developed.

## 1. Introduction

Buffaloes are widely distributed throughout the world, with Asia having the largest total number of buffaloes in stock [1]. It is reported that in 2020, the total world production of buffalo milk reached 134 million tons, accounting for 15.16% of the world’s total milk production, and becoming the world’s second-largest milk category after cow’s milk. During the five years from 2015 to 2020, the total world production of buffalo milk (15.86%) rose much more than that of cow’s milk (7.93%) (FAO 2020). Compared to cow’s milk, buffalo milk has a higher nutritional value, with a higher content of total solids (TS) (15.70 g/100 mL–17.20 g/100 mL), fat (5.30 g/100 mL–9.00 g/100 mL) and protein (2.70 g/100 mL–4.70 g/100 mL) [2,3], as well as minerals such as calcium and iron [4,5,6]. Additionally, probiotics such as lactic acid bacteria have a higher survival rate in buffalo milk, and buffalo milk has superior emulsification properties, making it very suitable for the production of chocolate milk, yogurt, cheese, and other dairy products [2,7,8]. Buffalo milk is also the world’s most famous traditional ingredient for cheese [9]. Khan et al. also suggested that buffalo milk has better antioxidant capacity and more health properties than cow’s milk [10]. Thus, buffalo milk has the potential to replace cow milk. Therefore, it is very necessary to establish a rapid detection technology for milk components such as milk fat, protein, and TS of buffalo milk, so as to provide a large number of phenotypic data for buffalo milk production and buffalo genetic breeding and provide a reference premise for buffalo milk production.

Predictive modeling based on mid-infrared spectroscopy (4000–400 cm^−1^, 2500–25,000 nm) enables rapid and non-destructive testing of batch samples, as well as a qualitative and quantitative characterization of complex biological samples and their components, including the analysis of compositional traits [11] and energy intake and detection of cow disease [12,13,14], dairy product quality [15], and adulteration [16,17,18]. The establishment of Fourier transform mid-infrared spectroscopy (FT-MIRS) at the end of the 1990s represented a major turning point in spectroscopic technology [19]. In 2012, FT-MIRS was approved by the International Committee for Animal Recording (ICAR 2012) as a standardized method for the analysis of milk components. Since the complete spectrum is a complex representation of the various organic components in the sample, pre-processing or selection of the obtained spectrum is necessary [20,21]. The methods of spectral variable selection are categorized into algorithmic selection and manual selection [21]. In terms of modeling algorithms, partial least squares (PLS) is currently the most commonly used method for developing applied equations, as it is simple and well established for generating new and complex features from infrared spectral data in different domains [21].

In terms of model validation and application, Ho et al., conducted an independent external validation based on different cow grazing years and found that the prediction accuracy of the model varied according to the year of grazing; the spring prediction was better than the spring prediction the following year when using the autumn samples as the training set [22]. Also, Macedo Mota et al., revealed that the relationship between the time at which the prediction sample was obtained and the time when the modeling sample was obtained had a certain influence on the prediction effect, specifically due to the use of old (2013) and new (2019–2020) data sets when interacting with the training and validation sets, although no significant differences were found. Moreover, differences in predictive modeling performance between farm-independent and randomized 10-fold cross-validation were observed [23], which contrasts with the conclusions drawn by Gabriel et al., who concluded that the predictions of group-independent external validation were similar to those of randomized validation [24]. Adkinson et al. also pointed out that different cross-validation strategies introduce varying levels of predictive deviation, with herd-independent cross-validation demonstrating the largest deviation, while cow-independent cross-validation yielded minimal deviations [25]. This indicates the influence of the spatio-temporal dimension on the prediction performance of the model. In particular, when newly developed models are applied, their performance can be compromised in the prediction of samples from different times and different farms due to the limitations of the sample collection time, the sample size, the representative and diverse samples, etc. Thus, the development and application of robust, ideal models necessitate the inclusion of a wide array of representative and diverse samples, along with a considerable time investment for model validation and refinement [26,27,28]. This highlights the urgent necessity for new methodologies to address the spatial and temporal challenges in predictive model implementation, ensuring that newly established models are adaptable and generalizable across diverse conditions.

Currently, the FT-MIRS models of cow milk are mature and widely used, whereas research focused on predictive models for buffalo milk based on FT-MIRS remains limited, primarily concentrated on adulteration detection and classification models [29,30]. Robust buffalo milk models have not been fully developed, and the rapid application of new models requires specific strategies. Therefore, to realize the rapid detection of buffalo milk composition, this study explored the prediction model for the content of milk constituents (milk protein, milk fat, and total solids) based on FT-MIRS of buffalo milk and its application strategies across two buffalo farms in southern and central China. The main objectives of this study were as follows: (1) to assess the feasibility of using FT-MIRS to establish buffalo-specific models for predicting the composition of buffalo milk and to establish an optimal quantitative model for buffalo milk based on FT-MIRS; (2) to evaluate the impact of temporal effects on the predictive performance of the model, thereby exploring the application strategy in the temporal dimension; and (3) to explore the application strategy of the model in the spatial dimension by examining the model’s performance across different farms.

## 2. Materials and Methods

### 2.1. Collection of Buffalo Milk Samples

A total of 655 buffalo milk samples were collected from August 2020 to July 2021 (excluding October 2020 and February 2021) in two buffalo farms in south China (farm A) and central China (farm B). The breeds included Mora, Nilafi, Mediterranean, and hybrid buffalo, with parities ranging from 1 to 6, a lactation period spanning from 5 to 400 days, and milk yield varying between 2.1 and 14.8 kg/d. The buffaloes were allowed to graze freely and were fed twice a day, with a diet primarily consisting of roughage, supplemented periodically with concentrated feed. The feed of farm A included elephant grass, peanut vines, corn stover, soybean meal, and cottonseed meal, whereas that of farm B included corn stover, soybean meal, bran, and canola. Each milk sample (80 mL) was collected in two sampling tubes, both spiked with a preservative (Bronopol, CAS 52-51-7). One tube was sent to the Dairy Herd Improvement (DHI) Measurement Center for FT-MIRS determination, while the second tube was used for chemical analysis of the buffalo milk composition.

### 2.2. Determination of FT-MIRS in Buffalo Milk

The determination of buffalo milk FT-MIRS was carried out according to the international standard “ISO 9622:2013 Milk and liquid milk products-Guidelines for the application of mid-infrared spectrometry: (1) milk samples were preheated in a thermostatic water bath at 45 °C for 30 min; (2) the milk FT-MIRS analyzer (MilkoScan^TM^ FT+, Denmark FOSS, Hillerød, Denmark) was cleaned and calibrated, with a wavelength range of 5011.54 cm^−1^ to 925.92 cm^−1^; (3) after thorough mixing, the samples were scanned using the FT-MIRS analyzer, and the corresponding FT-MIRS data were subsequently collected.

### 2.3. Screening of Milk Samples

The milk samples were screened based on 2 criteria: (1) the Mahalanobis distance of buffalo milk FT-MIRS (the Mahalanobis distance between each sample spectrum and the centroid of all sample spectra) was less than 3 [31], calculated by the following formula:

(1) $$D_{M}(x,y)=\sqrt{{\left(x-y\right)}^{T}S^{-1}(x-y)}$$

where x and y denote FT-MIRS from two samples, respectively, S is the covariance matrix, T denotes the transpose operation, and “−1” denotes the inverse matrix [32]; (2) the somatic cell count was less than 1000 K cells/mL. Finally, a total of 614 samples were selected for the chemical analysis and subsequent studies.

### 2.4. Determination of Reference Values for Milk Fat, Milk Protein, and Total Solids in Buffalo Milk

The milk fat content in buffalo milk was determined according to the fourth method (the Gerber method) in the national standard GB5009.6-2016 “National Food Safety Standard—Determination of Fat in Food”. The milk protein content in buffalo milk was measured by the third method (the combustion method) in the national standard GB5009.5-2010 “National Food Safety Standard—Determination of Protein in Food”. The total solids content in buffalo milk was determined by oven drying method according to the national standard GB5413.39-2010 “National Food Safety Standard—Determination of nonfat Milk Solids in Milk and Dairy Products”. The results of milk fat, milk protein, and TS content were expressed as percentages (%). Abnormal data, defined as those with |reference values—DHI values| > 3, were excluded. Finally, all samples remained. The final distribution of the samples is shown in Table 1. Reference values were analyzed using SPSS Statistics 26.0.

### 2.5. Modeling and Application

#### 2.5.1. Modeling

Sample data from September 2020 to March 2021 and May 2021 were used to build prediction models for milk fat, milk protein, and TS content of buffalo milk. The models’ performance was comparatively analyzed to identify the optimal quantitative model. Before the modeling, 10% of the sample data were randomly selected as part of an external validation set, which contains the data of the remaining months. The remaining 90% of the data, serving as a modeling set, were randomly divided into a training set and test set at a 4:1 ratio with 10-fold cross-validation applied during the modeling process. The spectral data were initially preprocessed using 12 different methods to eliminate the effects of particle size, surface scattered light, baseline interference, background noise or other unwanted noise, etc. These pre-processing methods included none, min–max scaling (MMS), standard scaler (SS), mean center (MC), standard normal variable transformation (SNV), moving average (MA), Savitzky–Golay (SG), first difference method (D1), second-order difference (D2), detrend correction (DT), multivariate scattering correction (MSC), and wavelet transform (WAVE) [33,34]. The selection of modeling feature bands was carried out by manual selection, wherein the full spectrum was randomly segmented into N parts and dynamically adjusted based on the previous research and the model training results. According to our laboratory’s previous research, the spectral regions associated with water absorption were retained in this study, as their inclusion is considered to improve the accuracy of the model. Partial least squares regression (PLSR) was used for all modeling algorithms. The optimal parameters of the models were sequentially determined based on the pre-processing methods and PLSR settings. The prediction performance of the model was evaluated using the coefficient of determination and root mean square error of the training, test, and validation sets (Rc^2^, Rp^2^, R_V_^2^; RMSE_C_, RMSE_P_, RMSE_V_) and the relative analytical error of the validation set (RPD_V_). The formulas for model evaluation metrics are as follows:

(2) $$bias={\left(\frac{\sum_{n=1}^{N}{\hat{y}}_{n}}{N}-\overset{‾}{y}\right)}^{2}$$

(3) $$R^{2}=1-\frac{\sum_{n=1}^{N}{\left(y_{n}-{\hat{y}}_{n}\right)}^{2}}{\sum_{n=1}^{N}{\left(y_{n}-\overset{‾}{y}\right)}^{2}}$$

(4) $$RMSE=\sqrt{\frac{\sum_{n=1}^{N}\left[{\left(y_{n}-{\hat{y}}_{n}\right)}^{2}\right]}{N}}$$

(5) $$RPD=\frac{{STD}_{EV}}{RMSE}$$

where y and ŷ represent the reference and predicted values, respectively. $\overset{‾}{y}$ is the mean value of y, N is the sample size, and STD_EV_ is the standard deviation of the samples [25,35].

The algorithms used in this study were all derived from the sklearn package of Python 3.10.

#### 2.5.2. Application of the Model to the Temporal Dimension

**Figure 1:**
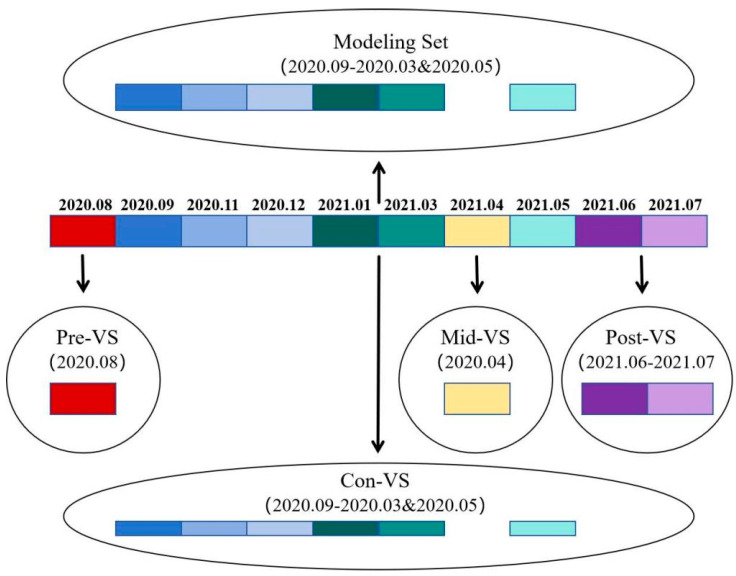
Partitioning structure of the data sets in the temporal dimension; the data sets with different colors represent different sampling months from 2020.08 to 2021.07. Note: Con-VS—Contemporaneous Period Validation Set; Mid-VS—Mid-period Validation Set; Ove-VS—Overall Validation Set; Post-VS—Post-period Validation Set; Pre-VS—Pre-period Validation Set.

As shown in Figure 1 and Table 2, all remaining data from August 2020, April 2021, and June–July 2021 were stratified into three different validation sets based on their temporal relationship with the sampling time of the modeling set. Data collected before the sampling time of the modeling set (August 2020) were assigned to the pre-period validation set (Pre-VS), data collected during the sampling time of the modeling set (not involved in the modeling, April 2021) were assigned to the mid-period validation set (Mid-VS), and data collected after the sampling time of the modeling set (June–July 2021) were assigned to the post-period validation set (Post-VS). In the above modeling procedure, some data were randomly selected for validation, coinciding exactly with the sampling time of the modeling set. This part was designated as the contemporaneous period validation set (Con-VS). All validation sets together were referred to as the overall validation set (Ove-VS). The optimal model above was applied to predict the samples of four different validation sets, namely, Pre-VS, Mid-VS, Post-VS, and Con-VS, and the R_V_^2^, RMSE_V_, and RPD_V_ of the four validation sets were measured to assess the predictive ability, the generalization ability, and the robustness of the model for each set, in order to investigate the temporal effects on the predictive effectiveness of the model.

#### 2.5.3. Application of the Model to the Spatial Dimension

**Figure 2:**
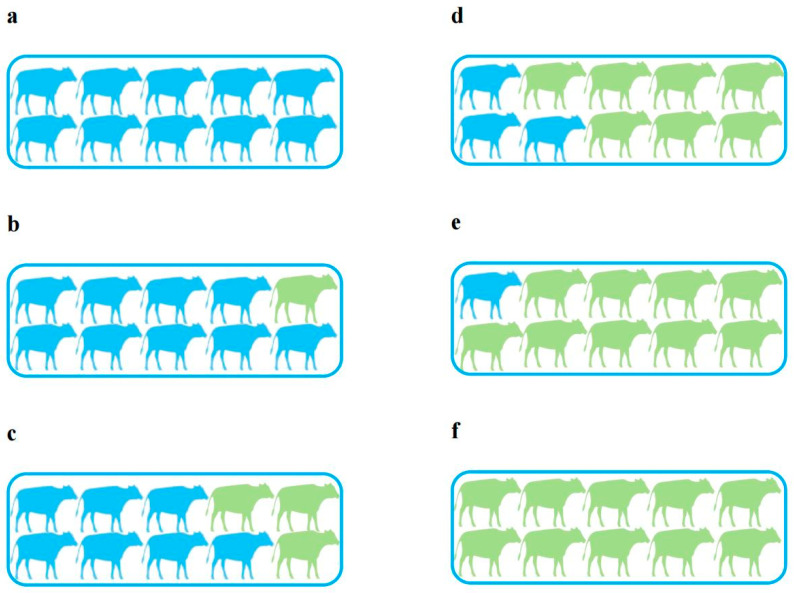
Partitioning structure of the modeling data sets in the spatial dimension, where blue represents farm A and green represents farm B. The proportion of buffalo in each group was estimated from the sample size, (**a**–**f**) represent groups 1 to 6, and the proportions of farm B in the modeling data are about 0%, 10%, 30%, 70%, 90%, and 100%.

As shown in Figure 2 and Table 3, the model of TS was initially developed only using 80% of the data from farm A, employing the same pre-processing method and modeling algorithm as the above optimal model. Then, the data from farm B (randomly selected from each sampling month according to the proportion) were sequentially added or the data from farm A were deleted to form new modeling data so that the six proportions of data from farm B among the total new modeling data were around 0%, 10%, 30%, 70%, 90%, and 100%.

During the modeling process, the data were randomly divided into a training set and a testing set at a ratio of 4:1, while the remaining data were designated as a validation set that included the farm A validation set, farm B validation set, and overall validation set of farm A and farm B. Consequently, six TS models were built using six different proportions of modeling data sets, following the same methodology as the optimal model described earlier. Each model was subsequently applied to predict the respective validation sets, and the predictive performance was evaluated using R_V_^2^, RMSE_V_, and RPD_V_ values.

## 3. Results

### 3.1. Predictive Models for Milk Fat, Milk Protein, and Total Solids Content

Table 4 shows the statistical situation of reference values of buffalo milk fat, milk protein, and TS. The mean levels of milk fat, milk protein, and TS in farm A were significantly higher than those in farm B (*p* < 0.05). However, the coefficient of variation for these components was lower in farm A compared to farm B.

**Figure 3:**
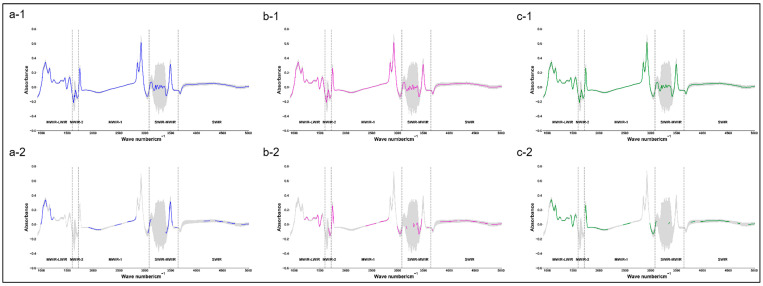
Comparison between the mean of the original spectrum and the mean of the spectrum after pre-processing + feature selection (under the optimal model) for buffalo milk. Note: The spectrum was mainly divided into five regions: Mid-Wave Infrared to Long-Wave Infrared (MWIR-LWIR), Mid-Wave Infrared 2 (MWIR-2, which is also the water absorption noise region), Mid-Wave Infrared 1 (MWIR-1), Short-Wave Infrared to Mid-Wave Infrared (SWIR-MWIR), and Short-Wave Infrared (SWIR); the curve and gray area in the figure represent the mean spectral value and SD of each wave point, respectively; (**a-1**,**a-2**) with blue color represent the mean ± SD of original FT-MIRS and the FT-MIRS after pre-processing (SG) + feature selection (302 wave points) in buffalo milk fat, and (**b-1**,**b-2**) with purple color represent the mean ± SD of original FT-MIRS and the FT-MIRS after pre-processing (SG) + feature selection (333 wave points) in buffalo milk protein, (**c-1**,**c-2**) with green color represent the mean ± SD of original FT-MIRS and the FT-MIRS after pre-processing (None) + feature selection (522 wave points) in buffalo milk TS.

Table 5 shows the optimal predictive models for buffalo milk fat, milk protein, and TS according to the model evaluation indicators. Figure 3 shows the comparison between the original mean spectra of buffalo milk fat, milk protein, and TS and the mean spectra obtained after pre-processing + feature selection (under the optimal model). The best predictive model for buffalo milk fat content was PLSR + SG (w = 15, *p* = 4) + 302 wave points, with R_C_^2^ of 0.78, R_P_^2^ of 0.77, R_V_^2^ of 0.82, and RPD_V_ of 2.39. The best predictive model for milk protein content of buffalo milk was PLSR + SG (w = 7, *p* = 4) + 333 wave points, with R_C_^2^ of 0.78, R_P_^2^ of 0.86, R_V_^2^ of 0.84, and RPD_V_ of 2.49. In addition, some characteristic wave points selected in the optimal model for milk fat and milk protein overlapped with the fat-absorption-related region (near the 1175.20 cm^−1^ region) [36] and protein-absorption-related region (near the 1541 cm^−1^ region) [37] of cow milk. The best predictive model for the TS content of buffalo milk was PLSR + None + 522 wave points, with R_C_^2^ of 0.87, R_P_^2^ of 0.86, R_V_^2^ of 0.86, and RPD_V_ of 2.64.

In a comparison of the predictive performance of the three optimal models, though the bias and RMSE_V_ of the TS model were not the lowest, the R_V_^2^ and RPD_V_ were the highest. Overall, the TS model demonstrated superior predictive performance compared to the milk fat and milk protein models. Furthermore, the RPD_V_ values of the three predictive models were greater than 2, indicating the potential application of the models in buffalo performance measurement. Therefore, to explore the accuracy and extensiveness of the predictive application of the model, this study selected the more effective TS model as an example and investigated the application strategy of the model across samples collected at different times and from different farms in both spatial and temporal dimensions.

### 3.2. Application of the Total Solids Content Prediction Model to the Temporal Dimension

Table 6 shows the predictive performance of the buffalo milk TS content predictive model in the validation set of different sampling periods as well as in the overall validation set. The results indicated that for the overall validation set, the model achieved a bias of 0.1283%, RMSE_V_ of 0.72, R_V_^2^ of 0.86, and RPD_V_ of 2.64. The most favorable performance was found in the contemporaneous period validation set, with the highest R_V_^2^ (0.92) and RPD_V_ (3.45) and the lowest RMSE_V_ (0.55). This was followed by the mid-period and post-period validation sets, with R_V_^2^ of 0.88 and 0.84, respectively, and the RPD_V_ all exceeding 2. The predictive performance of the pre-period validation set was weakest, with an R_V_^2^ of only 0.69, a higher RMSE_V_ of 1.01, and a lower RPD_V_ of less than 2. Overall, the predictive performance of samples from different sampling periods varied, which was shown in the order from the best to the worst: contemporaneous period > mid-period > post-period > pre-period. The model displayed better performance in predicting buffalo milk samples collected at the same time or at a nearby time in comparison to the modeled samples.

### 3.3. Application of Total Solids Content Prediction Models in the Spatial Dimension

The above results showed that the newly developed model can effectively predict the TS in buffalo milk samples collected at the same time or a nearby time in comparison to the modeled samples. However, it is necessary to explore the predictive performance and application strategy of the newly developed model when it is transformed and applied to buffalo milk samples collected from another farm (cross-farm).

Using the sample data of TS of buffalo milk from two different farms, the proportions of data accounted for by farm A and farm B in the modeling data set were calculated, and six predictive models based on different proportions of the modeling data set were developed and applied to different validation sets (farm A validation set, farm B validation set, and the overall validation set) for each group (Table 7).

As shown in Table 7, the predictive model developed based on one farm’s data had the best-developed performance for buffalo milk samples from the same farm (farm A or farm B). The farm A-based model (Group 1) showed superior performance in comparison to the farm B-based model (Group 6), with R_V_^2^ = 0.93 and RPD_V_ = 3.67 in the former and R_V_^2^ = 0.87 and RPD_V_ = 2.78 in the latter.

**Figure 4:**
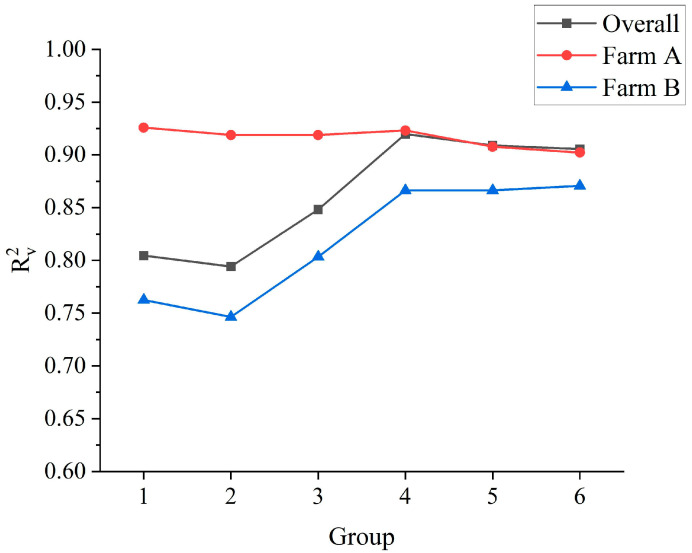
Trends of R_V_^2^ in the validation sets of different farms. Note: R_V_^2^—Coefficient of Determination of Validation Set.

When the farm A-based model was applied to farm B, as the proportion of farm B samples increased in the modeling (from 0% to 100%), the predictive performance of the model in the training set, the test set, and the validation set of farm A gradually decreased as indicated by a gradual decrease in R^2^ (R_C_^2^ from 0.93 to 0.74, R_P_^2^ from 0.96 to 0.80, and R_V_^2^ from 0.93 to 0.90) (Figure 4) and a gradual increase in RMSE (RMSE_C_ from 0.52 to 0.97, RMSE_P_ from 0.49 to 0.90, and RMSE_V_ from 0.50 to 0.63). Similar results were obtained when applying the farm B-based model to farm A. When the sample size ratio of farms A and B was approximately 3:7 (Group 4), compared to other groups, the model had stronger predictive performance in the training set, the test set, the overall validation set, and also the validation set of farm A and farm B, with RPD_V_ values all greater than 2.7 in farm A (3.61), farm B (2.74) and overall farms (3.53). Furthermore, even when the sample proportion of farm B was approximately 30% (Group 3), the model still had great predictive ability with RPD_V_ = 2.26, similar to farm A. Therefore, adding 30–70% of another farm’s data to the modeling data may realize the rapid transformation application of the model between farms.

### 3.4. Strategies for the Application of the Model for the Prediction of Total Solids Content

In the temporal dimension, the predictive model for TS content in buffalo milk demonstrated the best predictive performance when applied to samples collected during the contemporaneous period with the modeled samples, followed by the mid-period, with the lowest predictive effectiveness observed in the pre- and post-periods. In the spatial dimension, the optimal predictive performance was achieved when applying the model obtained by modeling data from one farm exclusively to predict that farm. In addition, if the one-farm-based model was applied to predict buffalo milk samples from other farms, incorporating a certain proportion (30–70%) of data from the predicted farm was required to debug the model.

A comprehensive analysis of predictive performance of model applications in both the temporal and spatial dimensions led to the following conclusions: (1) when applying the model to samples from a single buffalo farm, it is preferable to use a model built from sample data from this farm over the same period; (2) for cross-farm applications (applied to another farm), a certain proportion (30–70%) of sample data from another predicted farm can be added to the modeling data, preferably within the predicted time period, to debug the model, which can help to improve the accuracy of the model prediction; (3) to develop a robust model, for a single buffalo farm, long-term sample collection from the farm is essential, and for multiple farms or even a wider range, data should be collected at an appropriate proportion over a considerable period under the conditions of feeding and management of farms.

## 4. Discussion

### 4.1. Accuracy of the Model Based on FT-MIRS of Buffalo Milk for Predicting Three Milk Compositions

In this study, predictive models for milk fat, milk protein, and TS content in buffalo milk were developed using buffalo milk FT-MIRS, in which the best prediction model of TS content in buffalo milk was found with R_V_^2^ = 0.86 and RPD_V_ > 2, which may be related to the higher TS content in buffalo milk. The milk protein and milk fat models showed favorable performance but were inferior compared to the model for TS content prediction, with R_V_^2^ and RPD_V_ of 0.84 and 0.82, 2.49 and 2.39 for the two models, respectively. This may be due to the fact that milk fat and milk protein content in buffalo milk is lower than TS, and milk fat has a higher variability, being more readily decomposed into fatty acids and other products. Previous studies have reported that when RPD_V_ > 2, the model can be applied for purposes such as cow performance determination [38]. The R_V_^2^ of the predictive models for milk fat, milk protein, and total solids content in this study ranged from 0.82 to 0.86, and the RPD_V_ values were all greater than 2. This proves the feasibility of developing a buffalo-milk-specific milk composition predictive model based on buffalo milk FT-MIRS. However, some studies have reported that the R_V_^2^ of mid-infrared spectral models of cow milk fat and milk protein can reach 100% and 94%, respectively [39,40]. The R_V_^2^ of buffalo milk models in this study is lower, and further improvement is needed. Currently, PLS is widely used to build FT-MIRS models, so PLSR was applied in this study. However, some studies have suggested that the Bayesian algorithm may be superior to PLS in predicting milk composition and in technical characteristics [24,41]. Future research should explore alternative modeling algorithms to improve prediction accuracy. Additionally, the model’s predictive performance is influenced by the number of modeling samples, their diversity and representativeness, etc. Consequently, more samples are needed to optimize and improve the model in this study.

### 4.2. Strategies for Applying the Model in the Time Dimension

The predictive performance of the model for samples in different periods is different, following the order of the contemporaneous period > mid-period > pre-period and post-period. However, the prediction for the post-period is better than that of the pre-period, contrary to the findings of Ho et al., possibly due to the different components predicted [22]. We also propose the following possible reasons to explain the variation in predictive performance across different periods: (1) the contemporaneous validation set shares a temporal overlap with the training set, even though they are randomly selected and classified, leading to the highest spectral similarity; (2) the mid-period validation set is the data for the intermediate time period that does not overlap with the training set, but the modeling obtains the characteristics from both earlier and later periods. Therefore, the mid-period validation set has higher characteristic similarity than the pre-period and post-period validation set, which contributes to better predictive performance.

Therefore, we suggest optimizing the modeling by the following strategies when conditions fit: (1) when predicting a batch of samples, the predictive performance may be optimal using the model built based on the data completely overlapping with the time period of that batch; (2) on the basis of the existing model, adding some samples with the same period for modeling may help to improve the predictive performance of the model; (3) it is possible to lag the batch of samples and leapfrog to extract sample data from the previous and subsequent batches of the samples for modeling, which can improve the prediction accuracy for previous and subsequent batches of samples simultaneously and improves the accuracy in predicting dairy product components of the same batch, therefore reducing the workload of modeling and sampling; (4) also, this study illustrates that traits and characteristics may differ in each period. Establishing a model based on data spanning a sufficiently long time period can facilitate more comprehensive characteristics extraction, minimize period-specific biases, and improve the model’s generalizability.

### 4.3. Applying Strategies of the Model in the Spatial Dimension

This study revealed that the predictive performance of the TS model was superior when applied to buffalo milk samples from the same farm where the model was developed, compared to its application on an external farm. This is similar to the research of Tedde et al. on the body weight predictive model of cows [42] and the research of Chu et al. on the amino acid predictive models of cows [31]. However, there were some differences in the performance of the farm A-based model and farm B-based model in this study. Previous studies have reported that feed varieties and feeding practices [43,44,45], climate [46,47,48], etc., can affect the content of components and FT-MIRS in milk. The feeding management and production conditions between farm A and farm B in this study vary markedly: (1) Farm B is located in central China, with cold winters and hot summers, and the climate variation is larger than that of farm A. Buffaloes are susceptible to heat stress or cold stress. (2) Also, there are big differences in the feed types and production conditions between the two farms, with the feeding management of farm A being more systematic and stable. These variations may explain why the farm A-based total solids model demonstrated superior predictive performance in the validation set compared to the farm B-based model. This may be due to the farm B-based model extracting fewer effective characteristics related to TS content and having worse predictive performance, or the TS content of buffalo milk in the validation set of farm B having greater variability. This suggests that all the variations in different farms can lead to a great challenge in applying the model across farms, so this is the key problem that this study sought to overcome while proposing solutions and application strategies.

According to the results of this study, it was found that in the cross-farm application of the model, regardless of whether it is for farm A or farm B, the model application effect gradually reduced on the farm with the addition of data from another farm. However, the application effect on the other farm will gradually increase, and at the same time, the speed and magnitude of the application effect enhancement differed between farm A and farm B. These findings suggest that the following: (1) the model based on only the data of one farm is most suitable for itself, as the characteristics obtained by the model are also most suitable for that farm; (2) the model’s cross-farm application effect will be significantly improved with the inclusion of data from the other farm, and the characteristics of the model will change accordingly, so as to improve the model’s generalization ability; (3) the climate, the feeding management, and the production conditions of farm B are more varied than farm A. With the increase in data from farm B in modeling, the effect of cross-farm application of the model (prediction of the validation set of farm B) improves more obviously. Therefore, when the model is applied to another farm, it is better to add part of the data from the application farm. In addition, more sample data from the application farm may be needed when there are great variations in climate, feeding management, and production conditions.

Based on these findings, we suggest that it is critical to control the proportion of the data from application farms and thus to minimize the cost and improve the application effect of the model. In this study, the optimal farm A to farm B sample ratio was approximately 3:7, suggesting that (1) there may be an equilibrium point in the proportion of farm samples, and this equilibrium point is subject to the constraints of climate change, feeding management, and production conditions of the farm; (2) the proportion of samples from farm B is higher in the optimal model, which indicates that the model needs more samples to identify spectral characteristics for a farm with a large change in climate, feeding management, and production conditions to achieve better application effects; (3) for more in-depth studies in the future, the data set needs to be divided into more detailed parts and further analyzed in relation to the climate, feeding management, and production conditions of the farms to determine a better proportion of samples of predicted farms in the debugging of the model.

### 4.4. Comprehensive Analysis of the Models

This study demonstrated the feasibility of using FT-MIRS to predict the composition content of buffalo milk, with an application effect that can be improved by adopting certain strategies in the spatio-temporal dimension. Although the models developed in this study are not yet optimal, and the application strategies are not perfect, the findings provide valuable insights for future research and application of buffalo milk prediction models. Before the robust model is developed, the application strategy of the new model in spatio-temporal or other dimensions can be found to realize the rapid transformation and application of the models, so that a large-scale acquisition of phenotypic data of buffalo milk can be obtained, not only for TS of buffalo milk, but also for milk fat, protein, or more specific components, and potentially even for milk from other species. So, it can realize the herd management and genetic breeding analysis of buffalo, enrich the breeding target of buffalo, and realize the selective breeding of buffalo. Additionally, by accurately determining buffalo milk composition, producers can better control the selection and processing of buffalo milk products and produce more healthy or functional buffalo milk products for consumers. In addition, the findings of this study also provide a strategic reference for the development of robust models, further verifying that the robust model requires large, diverse, and representative sample data sets and takes a lot of time to develop [26,27,28].

## 5. Conclusions

In this study, predictive models for the milk fat, milk protein, and TS content of buffalo milk were developed based on FT-MIRS, all of which demonstrated good predictive performance (RPD_V_ > 2) within the sample range of this study. The predictive performance of the model for TS content was better than that of the predictive models for milk fat and milk protein content. Furthermore, this study explored application strategies for the model in both temporal and spatial dimensions. In the temporal dimension, the model exhibited superior predictions for samples collected in the contemporaneous period, followed by the middle and nearby post-period in comparison to the modeling sampling time. In the spatial dimension, the specialized model of one farm had the best predictive performance on the same farms. When applied across farms, incorporating an appropriate proportion (approximately 30–70%) of sample data from predicted farms into the model for debugging can improve the effect of the model’s application. However, there are some limitations in this study, such as a small number of participating farms and insufficient precision of data division in the spatial dimension. In the future, more sample data from more farms will be collected to optimize the predictive performance of the model. And the proportions of sample data from different farms will be further subdivided, and further analysis in combination with the feeding management and production conditions of the farms will be conducted. Factors such as the variability of the sample and spectrum will be addressed by means of spectral standardization, as well as parity and lactation stage corrections. Ultimately, a more accurate, extensive, and robust buffalo milk component predictive model will be developed, and the application strategy in the spatio-temporal dimension of the model will be perfected, in order to obtain large-scale phenotypic data for genetic analysis.

## Figures and Tables

**Table 1:** The quantity distribution of buffalo milk samples across farms and sampling times, after screening.

Trait	Farm	Sample Time										
		2020.08	2020.09	2020.11	2020.12	2021.01	2021.03	2021.04	2021.05	2021.06	2021.07	Total
All Samples	A	32	32	38	30	30	61	30	30	32	-	315
	B	5	10	-	34	-	108	27	34	63	18	299
Fat (%)	A	9	16	26	24	17	24	23	17	23	-	179
	B	4	8	-	24	-	88	-	31	62	18	235
Protein (%)	A	25	32	38	30	30	61	12	15	19	-	262
	B	5	10	-	34	-	48	27	34	62	18	238
TS (%)	A	23	32	38	30	30	59	30	23	19	-	284
	B	5	10	-	34	-	95	26	33	62	18	283

Note: Sample time indicates year and month. For example, 2020.08 indicates August 2020. TS—total solids.

**Table 2:** Application of the TS model in temporal dimensions.

Data Sets		Sample Size		
		Overall	Farm A	Farm B
Modeling set		347	191	156
Validation sets	Ove-VS ^1^	221	93	128
	Pre-VS ^2^	28	23	5
	Mid-VS ^3^	56	30	26
	Post-VS ^4^	99	19	80
	Con-VS ^5^	38	21	17

Note: Con-VS—Contemporaneous Period Validation Set; Mid-VS—Mid-period Validation Set; Ove-VS—Overall Validation Set; Post-VS—Post-period Validation Set; Pre-VS—Pre-period Validation Set. ^1^ All validation sets; ^2^ the sampling time of the validation set is before the sampling time of the modeling set; ^3^ the sampling time of the validation set is during the sampling time of the modeling set; ^4^ the sampling time of the validation set is after the sampling time of the modeling set; ^5^ the sampling time of the validation set coincides exactly with the sampling time of the modeling set.

**Table 3:** Application of the TS model in spatial dimensions.

Group	Modeling Set			Validation Set		
	Sample Size			Sample Size		
	Overall	Farm A	Farm B	Overall	Farm A	Farm B
1	228	228	0	339	56	283
2	256	228	28	311	56	255
3	314	228	86	253	56	197
4	313	86	227	254	198	56
5	255	28	227	312	256	56
6	227	0	227	340	284	56

**Table 4:** The statistical situation of reference values of buffalo milk fat, milk protein, and TS.

Trait	Farm	N	Min	Max	Mean ± SD	CV
Fat (%)	All Samples	414	1.77	13.7	7.27 ± 1.58	21.68%
	A	179	3.79	12.6	7.64 ± 1.55 ^a^	20.25%
	B	235	1.77	13.7	6.99 ± 1.54 ^b^	22.05%
Protein (%)	All Samples	500	2.84	7.2	4.73 ± 0.60	12.78%
	A	262	3.44	7.2	5.02 ± 0.53 ^a^	10.53%
	B	238	2.84	5.74	4.40 ± 0.51 ^b^	11.54%
TS (%)	All Samples	567	9.69	26.35	17.97 ± 2.07	11.49%
	A	284	10.6	26.35	18.62 ± 2.01 ^a^	10.81%
	B	283	9.69	24.41	17.33 ± 1.91 ^b^	11.04%

Note: N represents the sample size of buffalo milk, Min and Max represent the minimum and maximum values, respectively, reflecting the range of data; Mean ± SD represents the mean value ± standard deviation, reflecting the dispersion degree of data; CV represents the coefficient of variation, reflecting the variation degree of data. ^a,b^: Means having different superscripts within the same column are significantly different at *p* < 0.05.

**Table 5:** Global model evaluation results for milk fat, milk protein, and total solids.

Trait	Modeling Algorithm	Pre-Processing Method	Feature Number	Train Set		Test Set		Validation Set			
				R_C_^2^	RMSE_C_	R_P_^2^	RMSE_P_	Bias (%)	R_V_^2^	RMSE_V_	RPD_V_
Fat	PLSR	SG (w = 15, *p* = 4)	302	0.78	0.75	0.77	0.76	0.0181	0.82	0.65	2.39
Protein		SG (w = 7, *p* = 4)	333	0.78	0.26	0.86	0.23	0.1413	0.84	0.26	2.49
TS		None	522	0.87	0.75	0.87	0.88	0.1283	0.86	0.72	2.64

Note: Rc^2^—Coefficient of Determination of Train Set; RMSE_C_—Root Mean Square Error of Train Set; RMSE_P_—Root Mean Square Error of Test Set; Rp^2^—Coefficient of Determination of Test Set; Rv^2^—Coefficient of Determination of Validation Set; RMSE_V_—Root Mean Square Error of Validation Set; RPD_V_—Relative Analytical Error of Validation Set.

**Table 6:** The TS model predicting effects in the temporal dimensions.

Validation Set				
Group	Bias (%)	R_V_^2^	RMSE_V_	RPD_V_
Ove-VS ^1^	0.1283	0.86	0.72	2.64
Pre-VS ^2^	0.0002	0.69	1.01	1.80
Mid-VS ^3^	0.2495	0.88	0.67	2.87
Post-VS ^4^	0.5192	0.84	0.71	2.53
Con-VS ^5^	0.2707	0.92	0.55	3.45

Note: RMSE_V_—Root Mean Square Error of Validation Set; RPD_V_—Relative Analytical Error of Validation Set; R_V_^2^—Coefficient of Determination of Validation Set; Con-VS—Contemporaneous Period Validation Set; Mid-VS—Mid-period Validation Set; Ove-VS—Overall Validation Set; Post-VS—Post-period Validation Set; Pre-VS—Pre-period Validation Set. ^1^ All of the validation sets; ^2^ the sampling time of the validation set is before the sampling time of the modeling set; ^3^ the sampling time of the validation set is during the sampling time of the modeling set; ^4^ the sampling time of the validation set is after the sampling time of the modeling set; ^5^ the sampling time of the validation set coincides exactly with the sampling time of the modeling set.

**Table 7:** The predicting effects of the TS model in the spatial dimensions.

Group	Proportion of Sample Sizes in Farm A and B in Modeling Set		Train Set		Test Set		Validation Set											
							Overall				Farm A				Farm B			
	A	B	R_C_^2^	RMSE_C_	R_P_^2^	RMSE_P_	Bias (%)	R_V_^2^	RMSE_V_	RPD_V_	Bias (%)	R_V_^2^	RMSE_V_	RPD_V_	Bias (%)	R_V_^2^	RMSE_V_	RPD_V_
1	100.00%	0.00%	0.93	0.52	0.96	0.49	0.1730	0.80	0.88	2.26	0.9945	0.93	0.50	3.67	0.4838	0.76	0.93	2.05
2	89.06%	10.94%	0.93	0.54	0.93	0.59	0.3872	0.79	0.90	2.20	1.0727	0.92	0.52	3.51	0.9729	0.75	0.96	1.99
3	72.61%	27.39%	0.87	0.75	0.85	0.81	0.2432	0.85	0.77	2.57	1.6914	0.92	0.52	3.51	1.0060	0.80	0.83	2.26
4	27.48%	72.52%	0.79	0.94	0.89	0.67	0.3658	0.92	0.58	3.53	1.0864	0.92	0.56	3.61	0.8870	0.87	0.66	2.74
5	10.98%	89.02%	0.77	0.96	0.82	0.77	0.9304	0.91	0.63	3.31	1.9456	0.91	0.62	3.29	1.0045	0.87	0.66	2.73
6	0.00%	100.00%	0.74	0.97	0.80	0.90	1.3494	0.91	0.63	3.25	2.3006	0.90	0.63	3.20	0.4088	0.87	0.65	2.78

Note: Rc^2^—Coefficient of Determination of Train Set; RMSE_C_—Root Mean Square Error of Train Set; RMSE_P_—Root Mean Square Error of Test Set; RMSE_V_—Root Mean Square Error of Validation Set; Rp^2^—Coefficient of Determination of Test Set; RPD_V_—Relative Analytical Error of Validation Set; R_V_^2^—Coefficient of Determination of Validation Set.

## Data Availability

The original contributions presented in the study are included in the article, further inquiries can be directed to the corresponding authors.

## Abbreviations

The following abbreviations are used in this manuscript:

Con-VS	Contemporaneous Period Validation Set
CV	Coefficient of Variation
D1	First Difference Method
D2	Second-Order Difference
DHI	Dairy Herd Improvement
DT	Detrend Correction
FT-MIRS	Fourier Transform Mid-Infrared Spectroscopy
LWIR	Long-Wave Infrared
MA	Moving Average
Max	Maximum
Min	Minimum
MC	Mean Center
Mid-VS	Mid-period Validation Set
MMS	Min–Max Scaling
MSC	Multivariate Scattering Correction
MWIR	Mid-Wave Infrared
Ove-VS	Overall Validation Set
PLS	Partial Least Squares
PLSR	Partial Least Squares Regression
Post-VS	Post-period Validation Set
Pre-VS	Pre-period Validation Set
R_C_^2^	Coefficient of Determination of Train Set
RMSE_C_	Root Mean Square Error of Train Set
RMSE_P_	Root Mean Square Error of Test Set
RMSE_V_	Root Mean Square Error of Validation Set
R_P_^2^	Coefficient of Determination of Test Set
RPD_V_	Relative Analytical Error of Validation Set
R_V_^2^	Coefficient of Determination of Validation Set
SD	Standard Deviation
SWIR	Short-Wave Infrared
SG	Savitzky–Golay
SNV	Standard Normal Variable Transformation
SS	Standard Scaler
TS	Total Solids
WAVE	Wavelet Transform
